# Nourishing minds: the connection between healthy eating and academic success in higher education

**DOI:** 10.1186/s12889-026-26526-x

**Published:** 2026-02-07

**Authors:** Jillian Weathington, Emily King, Fatima Alziyad, Janaki Mutyala, Shanté Jeune

**Affiliations:** Department of Health Sciences, University of Central Florida, 12805 Pegasus Drive, HSII-215, Orlando, FL 32816 USA

**Keywords:** Diet quality, Academic performance, Higher education, Healthy eating index, Nutrition

## Abstract

**Background:**

Academic performance is often highly prioritized among college students, sometimes at the expense of their health. Despite growing interest in this relationship, limited research with college students has explored how diet quality (DQ) varies by gender, first-generation status, and grade-point average (GPA). The purpose of this paper was to: (1) examine the relationship between DQ and academic performance in college students and (2) identify potential differences based on gender, first-generation status, and varying GPAs.

**Methods:**

In this cross-sectional study, undergraduate students (*n* = 301), mean age 21.2 (SD ± 2.49), completed the validated Short Healthy Eating Index (sHEI) based on the USDA’s Healthy Eating Index (HEI) per *2015–2020 Dietary Guidelines for Americans*, to examine DQ. Academic performance was assessed using self-reported GPA. Students were predominantly non-Hispanic White (63%), Female (61%), and 75% had at least one parent graduate college. Descriptive statistics, correlation, and one-way ANOVAs were used to analyze the data using SPSS V.29. GPA was categorized into 3 groups: high, mid, and low GPA groups. Results were significant when *p* < 0.05.

**Results:**

DQ scores ranged from 21% to 68%, with a mean of 44% (SD: ±2.494). There were no significant associations between GPA and total DQ. However, significant associations were found between gender and specific dietary components. Further, total protein scores were greater among students with a high GPA compared to low and mid-GPA groups (F = 5.214, *p* = 0.006). Plant-based protein was greater among students who had at least one parent graduate college compared to first-generation students (F = 3.435, *p* = 0.034). Students living independently had lower total protein scores compared to those living with family (F = 4.841, *p* = 0.029). Additionally, students without a current job had higher dairy scores than those employed (F = 4.280, *p* = 0.039).

**Conclusion:**

Overall, college students reported poor DQ; however, personal (e.g., gender) and environmental factors (e.g., living arrangements) were associated with one’s DQ. Further investigation is needed to facilitate the development of effective interventions that encourage healthier dietary habits among college students to improve their overall health and wellness.

## Background

In 2024, there were approximately 15.3 million undergraduate students enrolled in college in the United States (US) [1]. Student success is primarily measured by one’s academic performance during university [2, 3]. However, as students pursue their academic careers, they are also burdened with transitioning into adulthood, adjusting to independent living, and learning to make their own lifestyle choices. Of the lifestyle choices, dietary behaviors can be adversely impacted as the student’s quest for success continues. In fact, national averages of diet quality (DQ) among emerging adults (ages 18–23) were 50.3%, based on the 2015–2020 Healthy Eating Index [4]. Consequently, these suboptimal dietary habits correlate with adverse health outcomes, including obesity, thereby increasing the susceptibility to non-communicable diseases such as type 2 diabetes and cardiovascular disease [5–7]. Because the college years are formative in establishing dietary patterns that often persist into adulthood, it is important to examine how dietary habits may influence students’ academic performance. However, DQ remains an often-overlooked factor when examining academic performance in college students.

The Social Cognitive Theory (SCT) provides a useful framework for understanding the factors that shape college students’ dietary behaviors. SCT proposes that health behaviors, including DQ, are derived from the interactions of personal, behavioral, and environmental factors, a concept known as reciprocal determinism [8]. The reciprocal relationship of these three factors, alongside key concepts, such as self-efficacy and self-regulation, work together to elicit behavior change [8]. Behavioral factors explain the actions an individual takes that can be influenced by their beliefs or environment [8]. Dietary behaviors of college students can fluctuate in response to various demands within college life. For some students, stress from their aspirations to perform well academically was associated with increased reliance on convenient, less healthy foods [7]. Whereas other students described making deliberate food choices as a form of self-care to support their health during stressful times [7]. These examples highlight how behavioral responses to environmental demands can vary, influencing DQ in both positive and negative ways.

Personal factors include the beliefs or characteristics of the individual that influence their behaviors, such as eating patterns and food choices [8]. Demographic characteristics, such as gender and first-generation status, can serve as individual factors that may alter dietary behaviors. Societal norms tied to gender may play an influential role in shaping motivations and attitudes towards healthy eating. Evidence suggests that males are more likely than females to report low motivation for healthy eating, lower enjoyment of healthy foods, and a tendency to prioritize other activities, such as hobbies, over maintaining a balanced diet [9]. Moreover, first-generation students often face greater difficulty in adapting to the demands of higher education, often navigating unfamiliar academic environments alone. Many also balance work and family responsibilities, which can contribute to heightened stress and a reduced capacity to engage in health-promoting behaviors, including maintaining better DQ [10]. As a result, the combined personal demands of stress, work, and responsibilities may pose challenges in achieving academic success. As such, DQ should be considered a key area of research among these subpopulations of college students.

Environmental influences, including the physical and social environments, can also make it more difficult for students to maintain healthy dietary habits. The college environment consists of increased exposure to readily available fast food and convenience items within and around campus settings [7, 11]. This food environment can create barriers to meal planning and preparation, leading many students, especially those with busy schedules and increased time demands, to rely more heavily on quick, convenient, and high-calorie food options [12]. Additionally, one’s social environment, particularly in living arrangements, may also shape students’ dietary behaviors. Prior research has suggested that students living independently were more likely to skip breakfast and consume sugary foods and beverages, compared to those living at home, who were more apt to consume meals more regularly, with reductions in overeating [13]. The interplay of the environment, personal, and behavioral factors demonstrates their reciprocal relationship, emphasizing how they collectively shape college students’ DQ.

Given the constructs of the SCT, the purpose of this study was to (1) examine the relationship between one’s personal characteristics, behavioral factors (DQ), and academic performance while in college (environmental), and (2) determine the between-group differences of one’s DQ (behavioral) by personal and environmental factors and varying GPA. It is hypothesized that better DQ among college students is associated with higher academic performance, whereas lower DQ is associated with lower academic performance. Additionally, it is hypothesized that there will be significant between-group differences of DQ, observed across varying levels of GPA, gender, and first-generation status. The literature has yet to fully examine how DQ may differ across varying levels of GPA and specific personal characteristics of students, such as gender and first-generation status, during their college years. Addressing this gap is essential for improving college students’ DQ by developing targeted approaches to encourage healthier eating habits across college campuses. A comprehensive understanding of the relationship between DQ and academic performance can offer valuable insight for developing effective interventions to help college students adopt and maintain healthier eating patterns, as well as guide the development of university-wide policies to improve DQ among students.

## Methods

### Participant recruitment and procedures

This study employed a cross-sectional design, conducted from September 2023 to April 2024. The study was conducted at a large, four-year public university located in the southeastern United States. The surrounding area is predominantly urban, with multiple grocery retailers located within two miles of campus; accordingly, the university is not classified as a food desert under the United States Department of Agriculture definition [14]. Despite the availability of nearby food resources, a 2024 campus survey indicated that 35% of students experienced food insecurity at this institution [15]. To help address this issue, the university operates a food pantry that provides food, clothing, and personal hygiene items to support student well-being, success, and retention. Students living both on and off campus have access to campus shuttle services and local bus lines, which may influence their ability to obtain groceries and fresh foods. The university also offers several dining halls and meal plan options, which may further affect students’ dietary behaviors and food access.

Participants were recruited for the study primarily through announcements in classrooms and student organizations held on campus, during which the researchers provided information and eligibility criteria to interested students. Additionally, informational recruitment flyers were posted in on-campus buildings and nearby student housing communities. Researchers also conducted in-person outreach at the university’s student union to speak to students individually and pass along informational flyers. Eligibility criteria for this study included currently enrolled, full-time, undergraduate students attending the university, aged 18 and older, and in junior or senior standing, including transfer students. Junior and senior students were selected to ensure GPAs reflect sustained academic performance and to capture more reliable dietary habits developed after the initial transition to college life. Exclusion criteria included college students under the age of 18, part-time college students, students previously diagnosed with an eating disorder, and students who take medication that affects appetite.

The primary purpose of this study was to examine the relationship between one’s personal characteristics, behavioral factors (DQ), and academic performance while in college. Based on a previous cross-sectional study among university students examining DQ and mental health outcomes, a small effect size of 0.2 was determined and utilized to assess their sample size [16]. Using the G-power software to calculate sample size, an effect size of 0.2 and a power of 0.8 requires a minimum sample size of 193 participants.

Interested participants were asked to scan a QR code on the recruitment flyer, taking them to an eligibility survey via Qualtrics. If determined to be eligible, participants received an online informed consent through Qualtrics, asking for consent to participate in the study. All participants provided informed consent prior to participating in any study procedures. After giving consent, participants completed validated questionnaires to examine demographics, DQ, and GPA. The online Qualtrics survey took participants approximately 20 min to complete. This study was approved by the university’s Institutional Review Board (IRB-23–00006090, October 17th, 2023) and the research was conducted in accordance with the Declaration of Helsinki.

### Study measures

#### Demographic measures

Demographic measures were collected to identify potential patterns across student subgroups and explore factors that may influence DQ and academic performance. Demographic variables collected in this study included age, race/ethnicity, gender, living situation, major, parent education level, number of enrolled credit hours, employment status, and employment hours worked per week.

#### Diet quality (DQ)

The Short Healthy Eating Index (sHEI) is a validated questionnaire used to examine DQ in adults to determine whether they meet the *Dietary Guidelines for Americans*. DQ refers to the extent to which an individual’s diet meets recommended levels of essential foods and nutrients necessary for maintaining overall health [17]. The sHEI was developed and validated by Colby et al. [18], has a low burden for participants, and is primarily used in studies with an online administration. The sHEI consists of 22 items and estimates consumption of 13 various components, including total fruits, whole fruits, total vegetables, greens and beans, whole grains, dairy, total protein, seafood and plant proteins, fatty acids, refined grains, sodium, added sugars, saturated fats, and total DQ. Internal consistency for the sHEI in the current sample was evaluated using Cronbach’s alpha, which demonstrated an acceptable reliability (α = 0.736). Previous studies examining DQ in college students have resulted in good validity and reliability of the sHEI [18–20].

#### Grade point average (GPA)

Students’ academic performance was determined through self-reported GPA. Self-reported GPA served as the metric for academic performance, given its established validity and positive correlation with actual GPA [21, 22]. For this study, the student’s current overall GPA was examined. For the purposes of this study, students were classified into three GPA categories: high, mid, and low, based on the university’s academic benchmarks to reflect meaningful distinctions in student performance: 3.0 as the minimum for major declaration, 3.8 as the threshold for graduating with honors, and 3.2–3.79 representing intermediate academic standing [23].

### Statistical analysis

Descriptive statistics, bivariate correlations, and one-way analysis of variance (ANOVA) analyses were used to analyze the data using SPSS V.29. For participant demographics, gender categories were classified as male and female. Participants were given the option to select non-binary or prefer not to say as responses for the gender category; however, sample sizes were too small to allow for meaningful statistical differences, and therefore these responses were excluded from analyses and solely reported in the descriptive table.

Bivariate correlation analyses were conducted to examine the possible associations between study variables. For the correlation analyses, GPA was treated as a continuous variable. One-way ANOVAs were conducted to determine the between-group differences of DQ among students with varying GPAs, personal factors (gender, first-generation status), and environmental factors (living arrangements, working status). For the ANOVA analyses, we categorized GPA groups into three groups: GPA of 3.8 and higher: high GPA; 3.2–3.79: mid-GPA; and 3.19 and below: low GPA. First-generation status was also classified into three categories: both parents graduated from college, one parent graduated from college, and neither parent graduated from college. Living arrangements were classified into two groups: students living independently from family and students living with family. Before conducting the one-way ANOVA, it was verified that its assumptions were met with independence of observations, normality of residuals using Shapiro-Wilk tests and Q-Q plots, and homogeneity of variances across groups using Levene’s test, all via SPSS. If there was significance between groups, a post-hoc Tukey Honestly Significant Difference (HSD) test was conducted to determine which groups were significantly different.

## Results

### Participant demographics

Study demographic characteristics can be found in Table 1. The sample included 301 undergraduate students. Students predominantly identified as non-Hispanic White (63%), female (61%), resided off campus (65.2%), lived with other students (57.3%), had a job (55.3%) with working hours of 11–20 h (24.5%), took 12–14 credit hours (62.6%), and 50% of students had reported both parents graduated college. The mean age was 21.2 years (SD: ±2.49). Most students were in a health-related major (24.8%), followed by engineering (13.2%), and communications (12.9%). These demographic results are comparable to national data of the college population [1, 24, 25]. Additionally, GPA among students ranged from 2.30 to 4.00 with a mean GPA of 3.48 (SD: ±0.391).

**Table 1:** Descriptive Statistics of Junior and Senior Standing College Students who completed Study Measures (*n* = 301).

		Sample	Percentage (%)
Race	White	191	63.2
	Black or African American	42	13.9
	American Indian or Alaska Native	1	0.3
	Asian	29	9.6
	Multiracial (two or more)	20	6.5
	Other	19	6.3
Gender	Male	111	36.8
	Female	185	61.3
	Non-binary/Third gender	4	1.3
	Prefer not to say	2	0.7
First-Generation Status	None	71	23.5
	One, mother only	38	12.6
	One, father only	40	13.2
	Both parents	152	50.3
Ethnicity	Hispanic	86	28.5
	Non-Hispanic	216	71.5
Job Status	Yes	167	55.3
	No	135	44.7
How many hours do you work?	0–10	28	9.3
	11–20	74	24.5
	21–30	52	17.2
	31–40	18	6.0
	41 or above	3	1.0
	I don’t currently have a job	127	42.1
How does your job affect your schoolwork?	I don’t have a job	135	44.7
	My job does not interfere with my schoolwork	50	16.6
	My job takes some time from my schoolwork	112	37.1
	My job takes a lot of time from my schoolwork	5	1.7
Residence	Dormitory	25	8.3
	Residence within walking distance of campus	76	25.2
	Residence within driving distance of campus	197	65.2
	Fraternity or Sorority house	4	1.3
Living Arrangements	Living alone	10	3.3
	With one or more students	185	61.3
	Spouse/Partner	8	2.6
	With child or children	2	0.7
	Parents	58	19.2
	Other relatives	10	3.3
	Other	29	9.6
Major	Agriculture	3	1.0
	Biological/life sciences	27	8.9
	Business	17	5.6
	Communication	39	12.9
	Computer and information sciences	17	5.6
	Education	4	1.3
	Engineering	40	13.2
	Health-related fields	75	24.8
	History	1	0.3
	Humanities	3	1.0
	Mathematics	3	1.0
	Multi/interdisciplinary studies	2	0.7
	Parks, sports management Physical sciences	2	0.7
	Pre-professional	22	7.3
	Public administration	3	1.0
	Social sciences	22	7.3
	Visual and performing arts	5	1.7
	Other	17	5.6
How many credits were you taking for the term?	1–3	1	0.3
	4–6	10	3.3
	7–11	24	7.9
	12–14	189	62.6
	15 or more	78	25.8

### Diet quality

Table 2 presents the mean score for total DQ and for the sHEI components among the sample. Among participants, DQ scores ranged from 21% to 68%, with a mean score of 44% (SD: ±2.49). Mean scores for individual components were also calculated, with each component scored according to its respective maximum value (out of 5 or 10 points). Among the components, the highest mean score was observed for total protein foods (4.75 out of 5) while the lowest mean score was for whole grains (3.12 out of 10), indicating variation in DQ across categories among our sample.

**Table 2:** Mean sHEI score and components

sHEI components	Maximum Points	Total Sample Mean (SD)
Adequacy		
Total Fruits	5	2.65 (± 1.91)
Whole Fruits	5	2.62 (± 2.09)
Total Vegetables	5	2.49 (± 0.76)
Greens and Beans	5	3.69 (± 2.20)
Whole Grains	10	3.12 (± 2.21)
Dairy	10	4.01 (± 1.14)
Total Protein Foods	5	4.75 (± 0.38)
Seafood and Plant Proteins	5	1.77 (± 1.33)
Fatty Acids	10	4.12 (± 1.14)
Moderation		
Refined Grains	10	4.29 (± 2.59)
Sodium	10	3.39 (± 2.14)
Added Sugar	10	3.33 (± 3.22)
Saturated Fats	10	3.89 (± 1.52)
Total sHEI score	100	44.1 (± 2.49)

**Fig. 1:**
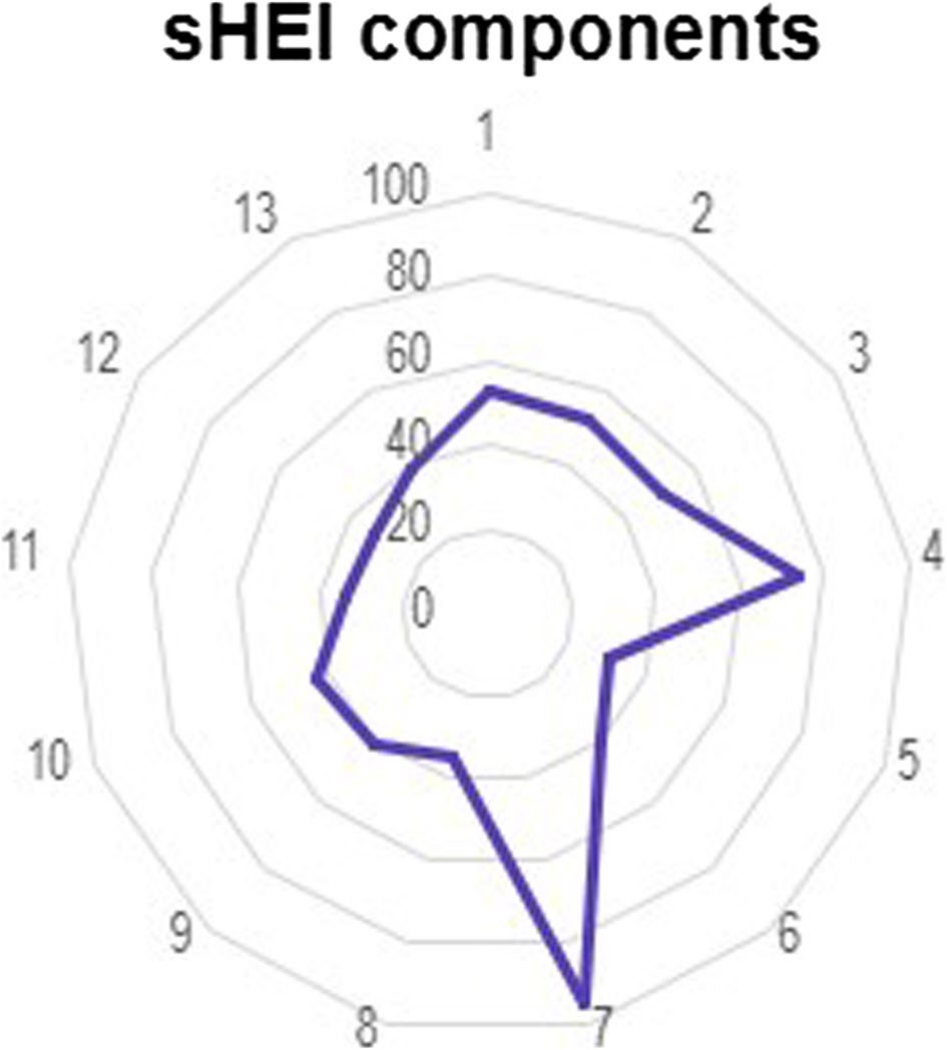
Radar chart of DQ scores across all study participants. Note: 1=total fruits, 2=whole fruits, 3=total vegetables, 4=greens and beans, 5=whole grains, 6=dairy, 7=total protein foods, 8=seafood and plant proteins, 9=fatty acids, 10=refined grains, 11=sodium, 12=added sugar, 13=saturated fats

Figure 1 displays the DQ radar chart. The DQ radar chart presents the average percentage-based scores for components of the sHEI, reflecting alignment with the *Dietary Guidelines for Americans*. Each axis represents a specific dietary component, scored from 0% (center of the chart) to 100% (outer edge). A score closer to the outer edge indicates better alignment to dietary recommendations, while scores near the center reflect poor alignment to dietary recommendations. Each component score in the DQ radar chart was calculated by dividing the mean score for that component by its maximum possible score, then multiplying by 100 to convert it to a percentage. The resulting percentages reflect how closely the average scores align with dietary recommendations for each category. For example, students in the sample scored high for total proteins (95%); however, students scored low for whole grains (31%).

#### Correlation matrix

Table 3 presents the data for the correlation matrix between measured variables, including means and standard deviations. No significant associations were found between GPA and total DQ; however, GPA was positively associated with greens and beans (*r*=0.118, *p* = 0.041), and total vegetables (*r*=0.141, *p* = 0.014). Females had significantly higher scores for whole grains (*r*=0.275, *p* < 0.001), dairy (*r*=0.155, *p* = 0.008), total protein (*r*=0.746, *p* < 0.001), plant proteins (*r*=0.350, *p* < 0.001), sodium (*r*=0.159, *p* = 0.006), and total DQ (*r*=0.168, *p* = 0.004) compared to males. Conversely, males had a significantly higher score for added sugar (*r*=−2.14, *p* < 0.001) compared to females.

**Table 3:** Correlation Matrix with Means and Standard Deviations of Demographics, Diet Quality, and GPA

Variables	1	2	3	4	5	6	7	8	9	10	11	12	13	14	15	16	17
1. Gender	1	0.005	0.051	0.168**	0.048	0.072	0.060	0.029	0.275**	0.155**	0.746**	0.350**	− 0.012	− 0.009	0.159**	− 0.214**	− 0.085
2. Age		1	− 0.164**	0.104	0.073	0.025	0.141*	0.118*	− 0.061	0.017	− 0.025	− 0.047	− 0.017	0.039	0.077	0.071	− 0.033
3. GPA			1	0.064	0.035	0.053	− 0.007	0.043	0.112	− 0.009	0.114	0.067	− 0.002	0.000	0.017	− 0.076	0.037
4. Total score				1	0.598**	0.650**	0.603**	0.581**	0.382**	0.159**	0.169**	0.458**	0.257**	0.605**	0.184**	0.465**	0.323**
5. Total fruits					1	0.829**	0.251**	0.227**	0.169**	0.124**	0.028	0.325**	0.079	0.171**	0.032	− 0.068	0.056
6. Whole fruits						1	0.252**	0.231**	0.190**	0.123*	0.007	0.298**	0.103	0.114*	0.011	0.170**	0.173**
7. Total vegetables							1	0.694**	0.148*	0.027*	0.098	0.232**	0.159**	0.566**	0.010	0.112	0.113*
8. Greens & Beans								1	0.179**	0.036	0.075	0.194**	0.073	0.498**	− 0.049	0.042	0.080
9. Whole grains									1	0.100	0.255**	0.220**	− 0.003	0.045	− 0.138*	− 0.031	0.066
10. Dairy										1	0.132*	0.212**	− 0.352**	− 0.031	0.023	− 0.092	− 0.017
11. Total proteins											1	0.356**	− 0.009	− 0.008	0.089	− 0.111	− 0.005
12. Seafood plant												1	0.012	0.202**	− 0.005	− 0.081	0.236**
13. Fatty acid													1	0.177**	− 0.018	0.243**	0.076
14. Refined grains														1	0.277**	0.153**	− 0.030
15.Sodium															1	− 0.056	− 0.398**
16. Added sugar																1	0.473**
17. Saturated fat																	1
Mean	**1.66**	**21.2**	**3.48**	**44.0**	**2.65**	**2.62**	**2.49**	**3.69**	**3.12**	**4.01**	**4.75**	**1.77**	**4.12**	**4.29**	**3.39**	**3.33**	**3.89**
Standard Deviation	**0.54**	**2.49**	**0.39**	**10.1**	**1.91**	**2.09**	**0.76**	**2.20**	**2.21**	**1.14**	**0.38**	**1.33**	**1.14**	**2.59**	**2.14**	**3.22**	**1.52**

"GPA" denotes grade-point average. * indicates *p* < 0.05; ** indicates *p* < 0.001

#### One-Way ANOVA analysis

##### Between-group differences with varying levels of GPA

One-way ANOVA analyses indicating significant differences in GPA groups are shown in Table 4. Significant differences were found for total protein (F = 5.214, *p* = 0.006) in groups with low and mid-GPAs, compared to high GPAs. Significant differences were also found for dairy (F = 3.460, *p* = 0.033) in groups with low and mid-GPAs, compared to high GPAs. No other significant differences were observed between GPA groups. A post-hoc analysis was conducted using the Tukey HSD method on statistically significant findings to identify where the significance occurred between groups. Total protein was found to be greater among students with a high GPA compared to both low (mean difference =−0.171, *p* = 0.018) and mid-GPA groups (mean difference =−0.145, *p* = 0.012). Although dairy was found to be significantly different overall among students with varying GPAs, the Tukey HSD post-hoc analysis could not detect significance between each of the individual groups.

**Table 4:** One-Way ANOVA Results between GPA and Diet Quality

Variable	Conditions	*N*	Mean Difference	*P*-value
Total Fruits	Low – High Mid – High Low – Mid	63 150 87	−0.925 −1.034 −0.426	0.233
Whole Fruits	Low – High Mid – High Low – Mid	63 150 87	−1.315 −1.124 −0.780	0.208
Total Vegetables	Low – High Mid – High Low – Mid	63 150 87	−0.269 −0.297 −0.188	0.736
Greens and Beans	Low – High Mid – High Low – Mid	63 150 87	−1.101 −0.668 −1.053	0.697
Whole grains	Low – High Mid – High Low – Mid	63 150 87	−1.562 −0.562 −0.921	0.097
Dairy	Low – High Mid – High Low – Mid	61 148 85	0.041 −0.330 −0.370	0.033*
Seafood and Plant Proteins	Low – High Mid – High Low – Mid	62 148 84	−0.254 −0.226 −0.028	0.392
Total Proteins	Low – High Mid – High Low – Mid	61 148 85	−0.171* −0.145* −0.026	0.006**
Fatty Acids	Low – High Mid – High Low – Mid	62 148 85	−0.044 0.219 −0.263	0.201
Refined Grains	Low – High Mid – High Low – Mid	63 150 87	−0.083 −0.166 0.083	0.892
Sodium	Low – High Mid – High Low – Mid	63 150 87	−1.084 −1.320 −0.362	0.071
Added sugar	Low – High Mid – High Low – Mid	63 150 87	−0.559 −0.398 −1.069	0.286
Saturated fats	Low – High Mid – High Low – Mid	63 150 87	−0.705 −0.348 −0.787	0.531
Total score	Low – High Mid – High Low – Mid	60 144 82	−6.403 −5.327 −3.999	0.279

One-way Anova results between GPA and diet quality. * indicates *p* < 0.05; ** indicates *p* < 0.001

##### Between-group differences with gender

One-way ANOVA analyses indicating significant differences in gender are shown in Table 5. Analyses showed female students had greater mean scores for whole grains (F = 14.19, *p* < 0.001), dairy (F = 4.826, *p* = 0.009), total proteins (F = 369.8, *p* < 0.001), added sugar (4.801, *p* = 0.003), and total DQ (F = 8.265, *p* = 0.004) compared to male students. Further, sodium mean scores were marginally higher among males compared to females (F = 2.608, *p* = 0.052).

**Table 5:** One-Way ANOVA results between Gender and Diet Quality

Variable	Conditions	*N*	Mean (± SD)	*P*-value
Total Fruits	Male Female	111 185	2.59 (± 1.99) 2.64 (± 1.86)	0.477
Whole Fruits	Male Female	111 185	2.47 (± 1.91) 2.66 (± 2.06)	0.508
Total Vegetables	Male Female	111 185	2.43 (± 0.76) 2.53 (± 0.76)	0.713
Greens and Beans	Male Female	111 185	3.60 (± 2.25) 3.73 (± 2.18)	0.528
Whole grains	Male Female	111 184	2.19 (± 1.14) 3.73 (± 2.47)	< 0.001**
Dairy	Male Female	110 185	3.82 (± 1.27) 4.11 (± 1.03)	0.009*
Seafood and Plant Proteins	Male Female	111 184	4.08 (± 1.79) 4.21 (± 1.84)	0.519
Total Proteins	Male Female	111 185	4.39 (± 0.41) 4.97 (± 0.00)	< 0.001**
Fatty Acids	Male Female	106 185	4.10 (± 1.19) 4.14 (± 1.12)	0.244
Refined Grains	Male Female	111 185	4.22 (± 2.59) 4.37 (± 2.61)	0.576
Sodium	Male Female	111 185	2.96 (± 2.17) 3.61 (± 2.09)	0.052
Added sugar	Male Female	111 185	4.14 (± 3.49) 2.91 (± 2.97)	0.003**
Saturated fats	Male Female	111 185	4.00 (± 1.51) 3.85 (± 1.51)	0.216
Total score	Male Female	105 183	41.8 (± 10.5) 45.3 (± 9.70)	0.004**

One-way Anova results between gender and diet quality.* indicates *p* < 0.05; ** indicates *p* < 0.001

##### Between-group differences with first-generation status

One-way ANOVA analyses indicating significant differences in first-generation status are shown in Table 6. Significant differences were found in seafood and plant-based proteins among first-generation students and those who had both parents attend college (F = 3.435, *p* = 0.034). A post-hoc analysis was conducted using the Tukey HSD method on the statistically significant findings to identify where the significance occurred between groups. First-generation students had significantly lower mean scores for seafood and plant protein (mean difference = −4.939, *p* = 0.027) compared to those who had both parents graduating from college.

**Table 6:** One-Way ANOVA Results between First-Generation Status and Diet Quality

Variable	Conditions	*N*	Mean Difference	*P*-value
Total Fruits	None – Both One – Both None – One	71 78 152	−0.497 −0.700 −0.515	0.767
Whole Fruits	None – Both One – Both None – One	71 78 152	−0.715 −0.965 −0.538	0.608
Total Vegetables	None – Both One – Both None – One	71 78 152	−0.187 −0.366 −0.109	0.318
Greens and Beans	None – Both One – Both None – One	71 78 152	−0.551 −1.138 −0.240	0.213
Whole grains	None – Both One – Both None – One	70 77 150	0.308 −0.054 0.362	0.550
Dairy	None – Both One – Both None – One	71 76 148	−0.206 −0.017 −0.188	0.437
Seafood and Plant Proteins	None – Both One – Both None – One	70 77 148	−0.494* −0.090 −0.403	0.034*
Total Proteins	None – Both One – Both None – One	70 76 149	0.038 0.124 −0.085	0.065
Fatty Acids	None – Both One – Both None – One	71 77 148	−0.470 −0.217 −0.688	0.410
Refined Grains	None – Both One – Both None – One	71 78 152	−1.016 −1.112 −0.881	0.766
Sodium	None – Both One – Both None – One	71 78 152	−0.432 −0.067 −0.365	0.364
Added sugar	None – Both One – Both None – One	71 78 152	0.001 −0.699 0.701	0.257
Saturated fats	None – Both One – Both None – One	71 78 152	−0.320 −0.293 −0.028	0.217
Total score	None – Both One – Both None – One	70 75 142	−0.816 −1.914 0.035	0.418

"None" denotes no parent. "One" denotes one parent. "Both" denotes two parents. * indicates *p* < 0.05; ** indicates *p* < 0.001

##### Between-group differences with environmental factors

One-way ANOVAs were also conducted on one’s environmental factors including the student’s living arrangement and working status. Students living independently were found to have reduced mean scores for total protein when compared to students who lived with family members (F = 4.841, *p* = 0.029). Additionally, students without a current job had greater mean dairy scores compared to those who were currently employed (F = 4.280, *p* = 0.039).

## Discussion

The findings of this study provide valuable insights into the associations among personal and environmental factors influential to students, DQ, and academic performance in college students. Contrary to our primary hypothesis, no significant associations were found between overall DQ and academic performance, suggesting that broader measures of DQ may not fully capture the associations of dietary patterns and academic outcomes. However, further analysis revealed significant variations in DQ across various subgroups, including GPA levels, personal characteristics such as gender and first-generation status, and environmental factors, including living arrangements and working status, were associated with differences in DQ. These findings are crucial for understanding the multifaceted factors that influence both diet and academic success in college students, and they offer a foundation for developing strategies aimed at improving DQ through targeted campus-based initiatives and policy changes.

Our study found no significant associations between academic performance and overall DQ, suggesting that GPA may not directly be influenced by students’ dietary habits. This aligns with prior research suggesting that while DQ is often considered an important factor for overall health, its direct impact on academic performance may not be as pronounced as other factors such as time management, stress, or even sleep quality [7]. Although GPA is commonly used as a measure of academic performance, it represents a cumulative outcome that may not fully capture the immediate academic stressors or environmental factors that may influence DQ. As such, its association with DQ may be more indirect compared to other factors such as time management or sleep quality; however, this has yet to be investigated. Despite the lack of correlation, participants’ average DQ was notably poor (44%). This may reflect common challenges identified in prior literature, such as time constraints, financial limitations, and limited awareness of healthy food options on campus, challenges that highlight the interplay of environmental conditions, personal factors, and behaviors described by SCT [26]. These shared barriers may explain the absence of significant associations between DQ and academic performance, as poor DQ appears to be prevalent among students, regardless of academic standing [27, 28].

Our study found that students with low and mid-GPAs had reduced total protein scores compared to those with high GPAs. This aligns with prior research linking increased consumption of plant-based proteins with better academic achievements [29]. However, it has been found that animal-based protein sources were inversely related to GPA outcomes [30]. This stratification is nuanced with complex outcomes in academic success. Within our study, animal-based protein was not measured as a sole category; we were, therefore, unable to determine whether specific protein sources differentially contributed to GPA differences. Future studies should aim to distinguish between plant- and animal-based protein contributions and explore potential mechanisms linking protein intake to academic outcomes.

Dairy was reduced among students with a low GPA, compared to those with a high GPA. This was a unique finding as previous research tracking self-reported GPA of university students alongside changes in diet found no significant change in GPA associated with milk consumption [31]. While dairy and GPA have not been widely studied in college populations, prior research among adolescents has found an association between milk intake and academic performance [32, 33]. Our study adds to this limited body of evidence by identifying a potential association in a college-aged sample. This finding is novel and warrants further investigation into how dairy influences academic performance in the college population.

It was found that there were significant differences among DQ and its components between males and females. Females in our sample had greater scores for whole grains, dairy, total proteins, and overall DQ, compared to males. Prior research indicates that women generally demonstrate greater nutritional awareness and are more proactive in food-related decisions, such as reading labels and selecting healthier options [25]. However, current literature on gender differences in DQ is not entirely consistent. For example, one study reported that males had higher quality scores for meat and dairy, while another found that males were more likely to prefer and regularly consume red meat [34, 35]. In contrast, our findings showed that females had higher quality scores for dairy and total proteins. Additionally, males in our sample exhibited a greater quality score for added sugar compared to females. Although existing research generally suggests that male college students consume more added sugar than females, these differences are not always statistically significant [36, 37]. Collectively, these findings underscore the complexity of gender differences in dietary patterns, thus highlighting the need for further investigation.

First-generation college students had reduced DQ in seafood and plant-based protein scores compared to their peers who had both parents graduating from college. Seafood and plant-based proteins are important sources of essential nutrients, and their lower frequency of consumption could be influenced by a combination of factors, including cultural dietary preferences, limited cooking skills, or gaps in nutritional knowledge [38]. According to data, 54% of first-generation students identify with a racial or ethnic minority group [39]. Therefore, it is important to consider cultural differences when discussing DQ. Cultural food preferences may not always be reflected in university dining offerings, which could make it challenging for some students, particularly first-generation college students, to access familiar, acceptable meals that also meet dietary guidelines for nutrient-dense foods. Moreover, prior research has highlighted that first-generation college students often face additional challenges, such as financial constraints, impacting their dietary choices [39]. Higher prevalences of food insecurity and reliance on lower-cost, less nutrient-dense options have been documented in this population, which could contribute to lower scores of seafood and plant-based proteins [40, 41]. While this study did not directly assess socioeconomic status (SES) or food access, these factors warrant consideration in interpreting dietary patterns. Future research should explore how financial constraints, food access, and cultural factors interact to shape DQ among first-generation college students.

Findings from our study suggest that college students residing with their families had greater total protein scores compared to those who lived independently, demonstrating that household food environments may play an important role in shaping dietary behaviors. Living independently requires students to manage both meal preparation and the associated costs [42]. The expense of protein-rich foods, such as meat, may therefore become a barrier to healthy eating among college students living on their own [42]. Recent studies examining nutrient intake by living arrangement among college students have reported no significant differences in protein intake between students residing in dormitories and those living at home [43]. However, earlier literature has observed reduced protein intake among students living independently, which aligns with our findings [44].

Further, college students who were employed (part-time and full-time) had lower dairy scores compared to students who were not employed. This finding is novel as there is limited literature examining the relationship between employment status and dairy among college students. A previous study among Korean university students found that all students, regardless of employment status, had low dairy consumption relative to national recommendations; however, differences in dietary guidelines and changes in recommendations over time limited direct comparisons [45]. Nonetheless, reduced dairy scores among employed college students highlights the role of environmental factors in shaping dietary behaviors among college students. Employment introduces more condensed time constraints and irregular schedules, which can increase reliance on convenience foods and limit opportunities for regular, nutrient-rich meals [46]. In contrast, living with family provides students with easier access to home-prepared meals, reducing the burden of meal planning and preparation, thus supporting the finding of greater overall protein scores [47]. These findings underscore how the physical and social environment can influence dietary choices among college students.

This study had several notable strengths. First, it included a sample size of 301 undergraduate students, enhancing the statistical power and generalizability of the findings. Recruitment strategies were intentionally designed to capture a diverse sample, including students from a wide range of academic disciplines. This approach ensures representation that reflects the broader college student experience. Additionally, the study contributes to a growing but still limited body of research examining DQ differences by first-generation status, an important and often underrepresented group in research related to nutrition and academic performance. Finally, the study design and measures are well-suited for replication in multicenter research across various institutional contexts, including public versus private and urban vs. rural colleges and universities.

While this study had several strengths, it is important to acknowledge its limitations. First, this study was a cross-sectional study; thus, a causal relationship cannot be established between DQ and academic performance. Future longitudinal or intervention-based studies could strengthen causal claims by examining changes in academic performance by following changes in DQ overtime. Second, self-reported GPA was used to assess one of the variables of interest in this study. Self-reported academic performance can be affected by social desirability, whereby participants report higher GPAs to appear more favorable, or self-serving distortions, where individuals overestimate their performance [48]. These biases could lead to misclassification of GPA groups and potentially influence the observed associations between GPA and dietary scores. While we did not verify GPAs through official records, previous research suggests that self-reported GPA is generally moderately to strongly correlated with official GPA, suggesting that any bias is likely to attenuate rather than exaggerate observed relationships [21, 22]. Lastly, the study did not explore cultural variations in diet. Although DQ was assessed based on whether students met the recommended *Dietary Guidelines for Americans*, culturally specific eating patterns (e.g., dietary restrictions) may not be fully captured by these guidelines, which could affect the generalizability of the findings. While this study didn’t explore specific cultural diets, this study provided a broad analysis of whether students generally met the recommended *Dietary Guidelines for Americans*. Future research may benefit from adapting dietary guideline-based measures to better reflect culturally diverse eating patterns.

## Conclusion

The findings of this study suggest that college students’ DQ was poor; however, it was not significantly associated with academic performance. Additionally, this research adds to the growing body of literature on DQ among college students by showing that, in this sample, both personal (gender & first-generation status) and environmental factors (living arrangements & employment) are associated with dietary behaviors. These results highlight the need for SCT-guided interventions to better understand how personal and environmental factors impact healthy eating behaviors among this population, to support both students’ dietary behaviors and academic outcomes. Although SES was not a central focus of this study, future research should also examine the influences of SES on nutrition awareness and eating patterns among college students. Overall, this study highlighted the role of DQ as a key behavioral factor influenced academic performance and the need for theory-based interventions to support student well-being and success.

## Data Availability

Data described in the manuscript, code book, and analytic code will be made available upon reasonable request from the corresponding author, S.J.
